# Analysis of ^13^C and ^14^C labeling in pyruvate and lactate in tumor and blood of lymphoma‐bearing mice injected with ^13^C‐ and ^14^C‐labeled pyruvate

**DOI:** 10.1002/nbm.3901

**Published:** 2018-02-19

**Authors:** E.M. Serrao, M.I. Kettunen, T.B. Rodrigues, D.Y. Lewis, F.A. Gallagher, D.E. Hu, K.M. Brindle

**Affiliations:** ^1^ Cancer Research UK Cambridge Institute University of Cambridge Cambridge UK; ^2^ Department of Biochemistry University of Cambridge Cambridge UK; ^3^ Department of Radiology University of Cambridge Cambridge UK; ^4^ A. I. Virtanen Institute for Molecular Sciences University of Eastern Finland Kuopio Finland; ^5^ Cancer Research UK Beatson Institute Glasgow UK

**Keywords:** hyperpolarized ^13^C, lactate, metabolism, pyruvate, tumor

## Abstract

Measurements of hyperpolarized ^13^C label exchange between injected [1‐^13^C]pyruvate and the endogenous tumor lactate pool can give an apparent first‐order rate constant for the exchange. The determination of the isotope flux, however, requires an estimate of the labeled pyruvate concentration in the tumor. This was achieved here by measurement of the tumor uptake of [1‐^14^C]pyruvate, which showed that <2% of the injected pyruvate reached the tumor site. Multiplication of this estimated labeled pyruvate concentration in the tumor with the apparent first‐order rate constant for hyperpolarized ^13^C label exchange gave an isotope flux that showed good agreement with a flux determined directly by the injection of non‐polarized [3‐^13^C]pyruvate, rapid excision of the tumor after 30 s and measurement of ^13^C‐labeled lactate concentrations in tumor extracts. The distribution of labeled lactate between intra‐ and extracellular compartments and the blood pool was investigated by imaging, by measurement of the labeled lactate concentration in blood and tumor, and by examination of the effects of a gadolinium contrast agent and a lactate transport inhibitor on the intensity of the hyperpolarized [1‐^13^C]lactate signal. These measurements showed that there was significant export of labeled lactate from the tumor, but that labeled lactate in the blood pool produced by the injection of hyperpolarized [1‐^13^C]pyruvate showed only relatively low levels of polarization. This study shows that measurements of hyperpolarized ^13^C label exchange between pyruvate and lactate in a murine tumor model can provide an estimate of the true isotope flux if the concentration of labeled pyruvate that reaches the tumor can be determined.

**Abbreviations used**
4‐CIN: α‐cyano‐4‐hydroxycinnamate
CSI: chemical shift image
EDTA: ethylenediaminetetraacetic acid
FDG: ^18^F‐fluorodeoxyglucose
HEPES: *N*‐2‐hydroxyethylpiperazine‐*N*′‐2‐ethanesulfonic acid
ID: injected dose
*k*_P_: apparent exchange rate constant for conversion of pyruvate to lactate
LDH: lactate dehydrogenase
MCT: monocarboxylate transporter
MRS: magnetic resonance spectroscopy
MRSI: magnetic resonance spectroscopic imaging
NADH: nicotinamide adenine dinucleotide
NMR: nuclear magnetic resonance
PET: positron emission tomography
SD: standard deviation
SEM: standard error of the mean
TCA: tricarboxylic acid
TE: echo time
TR: repetition time
TSP: 3‐(trimethylsilyl)‐2,2′,3,3′‐tetradeuteropropionic acid

## 1 INTRODUCTION

Metabolic imaging has long been used in the clinic, where it has been demonstrated to add value to standard anatomical imaging.^1^ The leading metabolic imaging technique, positron emission tomography (PET), when used with the glucose analog, ^18^F‐fluorodeoxyglucose (FDG), provides a measure of tissue glycolytic activity, which is frequently upregulated in tumors.^2^ FDG‐PET can be used to detect the presence of cancer, monitor disease progression and detect tumor responses to treatment.^1^, ^3^ The introduction of hyperpolarized ^13^C‐labeled cell substrates has enabled a new approach to metabolic imaging. Nuclear spin hyperpolarization of ^13^C‐labeled molecules can increase their sensitivity to detection in the ^13^C nuclear magnetic resonance (NMR) experiment by more than 10 000‐fold.^4^ Intravenous injection of hyperpolarized ^13^C‐labeled cell substrates and subsequent ^13^C magnetic resonance spectroscopic imaging (MRSI) enable the real‐time imaging of tissue metabolism in the clinic, in tumors^5^ and in the heart.^6^ Hyperpolarized [1‐^13^C]pyruvate is the best‐established substrate, mainly as a result of its relatively long polarization half‐life (~30 s *in vivo*), rapid plasma membrane transport and subsequent metabolism.^7^, ^8^ Pyruvate is well tolerated at relatively high concentrations and has a history of safe use in humans as well as in animals.^5^ Under pathological conditions that lead to upregulated glycolysis, such as in the majority of tumors, pyruvate, which is the end product of glycolysis, is not oxidized, but is rapidly and reversibly reduced by nicotinamide adenine dinucleotide (NADH) to generate lactate, in a reaction catalyzed by lactate dehydrogenase (LDH). Similarly to FDG‐PET, MRS(I) measurements of hyperpolarized ^13^C label exchange between injected [1‐^13^C]pyruvate and endogenous lactate have been used in cancer to detect disease and monitor disease progression,^9^, ^10^ and to assess early tumor responses to treatment.^11^, ^12^ The experiment has an advantage over FDG‐PET in that it does not involve the use of ionizing radiation, which could allow multiple repeat measurements in an individual patient, for example to guide treatment, and it assesses a unique metabolic feature of tumors, i.e. their preference to reduce pyruvate to lactate rather than oxidize it in the mitochondrial tricarboxylic acid (TCA) cycle. FDG‐PET measures glucose uptake and subsequent phosphorylation, which can be upregulated in normal tissues, such as the brain, as well as in tumors.

^13^C labeling of the endogenous lactate pool following the injection of hyperpolarized [1‐^13^C]pyruvate is often characterized by the [1‐^13^C]lactate/[1‐^13^C]pyruvate signal ratio or by an apparent first‐order exchange rate constant if the metabolite signals are analyzed as a function of time.^7^, ^8^, ^11^ However, as the concentrations of labeled pyruvate and lactate cannot be determined from their signal intensities, it is not possible to determine a metabolically relevant flux, for example, in mM/s.^13^ This is a problem as changes in this apparent first‐order rate constant could be the result of a metabolically relevant change in isotope flux, resulting, for example, from a change in LDH concentration or a change in the concentration of pyruvate that actually reaches the exchange site. This is not a significant problem for preclinical studies as, typically, inbred strains of mice are used and, in the case of tumors, nearly identical tumors are implanted, or autochthonous tumors arise at the same site, and therefore variations in pyruvate delivery are less likely. However, this is likely to be much more of a problem in the clinic.

Here, we have used measurements with [1‐^14^C]pyruvate to show that less than 2% of the injected pyruvate reaches the tumor site. Estimates of this tumor pyruvate concentration, together with the apparent first‐order rate constant describing ^13^C label exchange between injected hyperpolarized [1‐^13^C]pyruvate and the endogenous lactate pool, were used to estimate isotope label flux. This showed good agreement with the flux estimated directly from measurements of [3‐^13^C]lactate concentrations in extracts of tumors from animals injected with [3‐^13^C]pyruvate. Injection of hyperpolarized [1‐^13^C]pyruvate into animals treated with a lactate transport inhibitor and injected with a gadolinium chelate, and analysis of changes in the hyperpolarized [1‐^13^C]lactate signal intensity, showed that there was significant export of labeled lactate from the tumor cell *in vivo*. The contribution of any lactate that is labeled elsewhere in the body and washes into the tissue of interest has previously been assumed to be negligible as imaging studies with hyperpolarized [1‐^13^C]pyruvate in this and other tumor models have shown the presence of only low levels of labeled lactate in the blood pool.^11^, ^14^, ^15^ Here, we examined this assumption in more detail and showed that there are significant levels of labeled lactate in the blood at 30 s after injection of labeled pyruvate, determined by measurement of the thermally polarized ^13^C in tissue extracts. However, ^13^C imaging measurements performed with hyperpolarized [1‐^13^C]pyruvate in this study and previously^14^ show no evidence for the appearance of labeled lactate, suggesting that it has only relatively low levels of polarization.

## 2 METHODS

### 2.1 Tumor implantation

All animal experiments were conducted in accordance with project and personal licenses issued under the UK Animals (Scientific Procedures) Act, 1986. Protocols were approved by the Cancer Research UK, Cambridge Institute Animal Welfare and Ethical Review Body.

Tumors were established by subcutaneous inoculation of a suspension of 5 × 10^6^ viable EL4 murine lymphoma cells (in a volume of 100 μL) in the left flank of C57BL/6 mice (Charles River Ltd., Harlow, Essex, UK) (*n* = 69) at 6–8 weeks of age, when the animals were ~20 g. Studies were performed when the tumors had grown to a size of ~2 cm^3^, which was typically 10 days following implantation. Animals, which were not submitted to any dietary restriction,^16^ were anesthetized by either inhalation of 1–2% isoflurane (Isoflo, Abbotts Laboratories Ltd., Maidenhead, Berkshire, UK) in air/O_2_ (75%/25%, 2 L/min) or by intraperitoneal administration of 10 mL/kg body weight of a 5: 4: 31 mixture of Hypnorm (VetaPharma, Sherburn‐in‐Elmet, Leeds, North Yorkshire, UK), Hypnovel (Roche, Welwyn Garden City, Hertfordshire, UK) and saline (*n* = 5). Body temperature was maintained by blowing warm air through the magnet bore. The breathing rate (~80 beats/min) and body temperature (37°C) were monitored during the experiments (Biotrig, Small Animal Instruments, Stony Brook, New York, USA). Agents were injected intravenously via a tail vein catheter.

For some experiments (*n* = 6), the transport of hyperpolarized [1‐^13^C]pyruvate and [1‐^13^C]lactate was inhibited by injecting a monocarboxylate transporter (MCT) inhibitor, α‐cyano‐4‐hydroxycinnamate (4‐CIN, 150 mg/kg, 0.2 mL intravenously),^17^ 3 min (*n* = 2) and 30 min (*n* = 4) prior to the injection of hyperpolarized [1‐^13^C]pyruvate (10 mL/kg, 82mM). There was no difference between these two groups of animals in the results obtained (data not shown).

### 2.2 Hyperpolarization of [1‐^13^C]pyruvate

Pyruvate was hyperpolarized as described previously.^4^ Briefly, [1‐^13^C]pyruvic acid samples (44 mg, 14 mol/L; 91% ^13^C) containing 15 mmol/L of trityl radical, tris(8‐carboxy‐2,2,6,6‐tetra‐(hydroxyethyl)‐benzo‐[1,2‐4,5]‐bis‐(1,3)‐dithiole‐4‐yl)‐methyl sodium salt (OX063; GE Healthcare, Amersham, Buckinghamshire, UK) and 1.5 mmol/L gadolinium chelate (Dotarem, Guerbet Laboratories Ltd, Solihull, West Midlands, UK) were polarized using a microwave source at 93.982 GHz and 100 mW for 1 h in a 3.35‐T Hypersense (Oxford Biotools, Abingdon, Oxfordshire, UK) polarizer. The frozen sample was then dissolved at 180°C in 6 mL of buffer containing 40mM *N*‐2‐hydroxyethylpiperazine‐*N*′‐2‐ethanesulfonic acid (HEPES), 94mM NaOH, 30mM NaCl and 50 mg/L ethylenediaminetetraacetic acid (EDTA). Polarization levels ranged from 16% to 25%, measured using a polarimeter (Oxford Instruments, Abingdon, Oxfordshire, UK).

### 2.3 MRS and MRSI

Experiments were performed in a 7.0‐T horizontal bore magnet (Agilent, Palo Alto, CA, USA) using an actively decoupled dual‐tuned ^13^C/^1^H volume transmit coil (Rapid Biomedical, GmbH, Rimpar, Germany; inner diameter, 42 mm) and a 20‐mm‐diameter ^13^C surface receiver coil (Rapid Biomedical) placed over the tumor. In order to localize the tumor, transverse ^1^H images were acquired using a spin‐echo pulse sequence [repetition time (TR), 1.5 s; echo time (TE), 10 ms; field of view, 40 mm × 40 mm; data matrix, 128 × 128 points; slice thickness, 2 mm; 15 slices]. Hyperpolarized [1‐^13^C]pyruvate (10 mL/kg, 82mM) was injected intravenously during data acquisition. Single transient spectra from a 6‐mm‐thick tumor slice were collected using a slice‐selective excitation pulse with a nominal flip angle of 10° (*n* = 15). One hundred and eighty ^13^C spectra were acquired with a TR of 1 s. In some of these experiments (*n* = 10), a gadolinium chelate [300 μL of 0.05 mmol/mL (~0.75 mmol/kg) Prohance, Bracco, Italy] was injected as a bolus via a tail vein catheter over a period of 2–3 s, 30–40 s after the start of injection of hyperpolarized [1‐^13^C]pyruvate, as described previously.^18^, ^19^ The data were analyzed in Matlab (The Mathworks, Massachusetts, USA). The integrated peak intensities of hyperpolarized [1‐^13^C]pyruvate and [1‐^13^C]lactate were fitted to a simple product model,^20^ where *k*_P_ is a first‐order rate constant describing the labeling of lactate (L) from pyruvate (P) and *R*_1,L_ describes the loss of polarization in lactate as a result of spin–lattice relaxation (R_1,L_):

(1) $$\frac{dL}{dt}=k_{P}P-\left(R_{1,L}+R_{1,\mathrm{Gd}}\right)L$$

To account for the effect of Prohance, a second relaxation term (*R*_1,Gd_) was included in the model at time points after Prohance injection (30–40 s). To avoid any bias arising from this approach, in animals not receiving Prohance (*n* = 5), *R*_1,Gd_ was incorporated into the model at 35 s after pyruvate injection, which yielded a value for *R*_1,Gd_ of 0.001 ± 0.001 s^–1^.

In another set of animals (*n* = 2), axial ^13^C chemical shift images (CSIs) (TR = 30 ms; TE = 1.5 ms; field of view, 40 mm × 40 mm; data matrix, 24 × 24 with center‐out phase encoding order; spectral width, 6 kHz; total acquisition time, 30 s; flip angle, 5°) were acquired from an 8‐mm‐thick slice, which was selected from the ^1^H images. Images were acquired 15 s after injection of [1‐^13^C]pyruvate, using a volume coil in transmit/receive mode. The data were multiplied by a cosine function and zero‐filled to 128 points in both spatial directions, line‐broadened to 20 Hz and zero‐filled to 1024 points in the spectral dimension before Fourier transformation, phase and baseline correction and peak integration.

### 2.4 Tissue and blood collection and analysis

#### 2.4.1 ^13^C experiments

Perchloric acid extracts of blood and tumor tissue from EL4 tumor‐bearing (*n* = 7) and non‐tumor‐bearing (*n* = 6) mice were prepared from animals that had been anesthetized for 30 min and then injected with [3‐^13^C]pyruvate. Thirty minutes corresponds to the duration of the studies with hyperpolarized [1‐^13^C]pyruvate. Thirty seconds after injection of [3‐^13^C]pyruvate (10 mL/kg, 82mM), blood was obtained by cardiac puncture and the mice were sacrificed by cervical dislocation, followed by rapid excision and freeze‐clamping of tumors in liquid nitrogen‐cooled tongs. Blood was collected into sodium fluoride‐coated tubes (BD Microtainer, Oxford, UK) and centrifuged (18 800 ***g***) at 4°C for 2 min. Plasma was then added to another Eppendorf tube with 600 μL of 7% perchloric acid (1: 8, w/v). In another cohort of mice (*n* = 3, tumor‐bearing; *n* = 4, non‐tumor bearing), blood was withdrawn by cardiac puncture and 0.4 mL was added to a tube preheated to 37°C to which [3‐^13^C]pyruvate (40 μL, 82mM) was then added rapidly and the tube was gently mixed. After 30 s, the blood samples were processed as above. In another set of animals (*n* = 5), anesthetized by intraperitoneal injection of Hypnorm/Hypnovel, [1‐^13^C]lactate (45mM)^21^ was injected intravenously into tumor‐bearing animals that had received either saline (*n* = 3) or 4‐CIN (*n* = 2) injections 5 min prior to lactate injection. Animals were killed 30 s later and the tumors were processed as above.

Perchloric acid extracts were prepared using ice‐cold 7% perchloric acid (1: 8, w/v), which were then neutralized with KOH, lyophilized and dissolved in 99.9% deuterium oxide. The experiments in which [3‐^13^C]pyruvate was added to samples of blood *ex vivo* showed that, following this perchloric acid extraction procedure, ~90% of the added ^13^C label was recovered in pyruvate and lactate.

High‐resolution ^1^H and ^1^H‐decoupled ^13^C NMR spectra of plasma and tumor were obtained at 14.1 T (25°C, pH 7.2) using a 600‐MHz NMR spectrometer (Bruker, Ettlingen, Germany) and a 5‐mm probe. The acquisition conditions were as follows: ^1^H, 90° pulses; spectral width, 7.3 kHz; acquisition time, 4.5 s; 32 k data points; 64 transients; recycling time, 12.5 s; ^13^C, 30° pulses; spectral width, 36.0 kHz; acquisition time, 0.9 s; 32 k data points; 2048 transients; recycling time, 14 s. Chemical shifts were referenced to 3‐(trimethylsilyl)‐2,2′,3,3′‐tetradeuteropropionic acid (TSP, 0.0 ppm). Spectral deconvolution and multiplet structures were analyzed using the PC‐based (Intel Centrino Platform) NMR program, ACDSpecManager (ACD/Labs, Bracknell, Berkshire, UK). Data were zero‐filled twice and multiplied by an exponential function prior to Fourier transformation. All NMR resonance areas were normalized to the integral of the 5mM TSP resonance.

#### 2.4.2 ^14^C experiments

[1‐^14^C]Pyruvate, with a specific activity of 50–60 mCi/mmol, was supplied by American Radiolabeled Chemicals (Royston, Hertfordshire, UK). An 82mM pyruvic acid solution containing 200 kBq ^14^C was injected per animal. The ^14^C pyruvic acid was mixed with cold pyruvic acid in order to achieve the same pyruvate concentration as that used in the imaging experiments (10 mL/kg, 82mM).

Fifteen tumor‐bearing mice were maintained under anesthesia for 30 min (the duration of the hyperpolarized ^13^C studies) prior to injection with [1‐^14^C]pyruvate. Animals were killed at 15, 30 and 60 s after injection and the blood and organs were removed. The organs were homogenized in RIPA buffer (1: 5) (50mM HEPES, 1mM EDTA, 0.7% sodium deoxycholate, 1% Nonidet P‐40, 0.5 M lithium chloride, pH 7.6) using a Precellys 24 homogenizer (Stretton Scientific Ltd., Stretton, Derbyshire, UK) and the blood was centrifuged as described above. The counts from the tissues and serum were determined using a liquid scintillation counter (Perkin Elmer, Beaconsfield, Buckinghamshire, UK). The measured radioactivity was normalized to tissue weight or serum volume, accordingly.

### 2.5 Statistical analysis

Data are reported as the mean ± standard error of the mean (SEM). Statistical significance was tested using Prism 6 with a two‐tailed Mann–Whitney test, unpaired two‐tailed Student's *t*‐test or Kruskal–Wallis test when appropriate. *p* < 0.05 was considered to be significant.

## 3 RESULTS

### 3.1 Biodistribution of pyruvate

**Figure 1:**
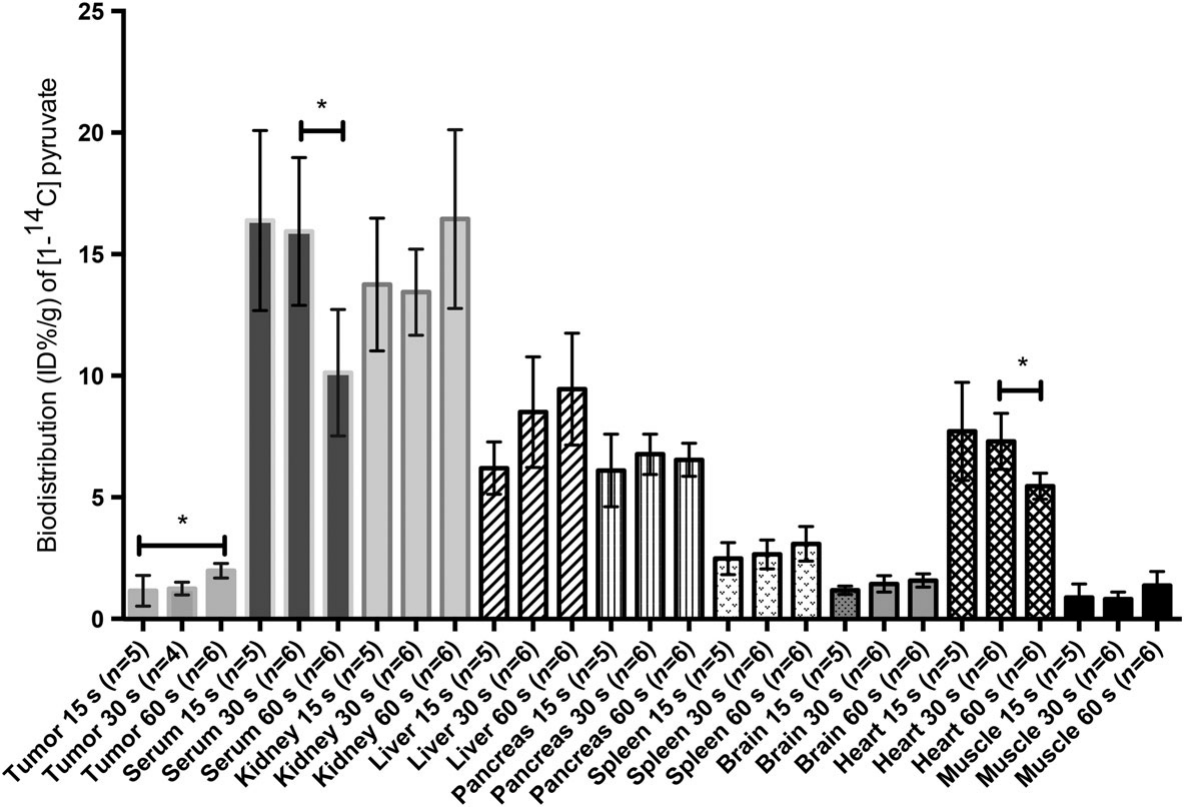
Biodistribution [percentage of injected dose (ID%)/g] of [1‐^14^C] pyruvate injected intravenously (10 mL/kg; 82mM; specific activity, 50–60 mCi/mmol). EL4 tumor‐bearing mice were injected at the indicated times and the tissues were harvested and weighed, and the radioactivity was counted in a scintillation counter. The total amounts of radioactivity recovered from the indicated tissues were 55.9, 57.9 and 56.1 ID%/g at 15, 30 and 60 s, respectively

The biodistribution of [1‐^14^C]pyruvate (10 mL/kg; 82mM; specific activity, 50–60 mCi/mmol) in EL4 tumor‐bearing mice was studied at 15, 30 and 60 s after intravenous injection (Figure 1). Only a small fraction of the injected [1‐^14^C]pyruvate was observed in the tumor, with a significant increase occurring between 15 [1.16% injected dose (ID)/g tissue] and 60 s (1.98% ID/g). ^14^C levels in the plasma were ~15% ID/g and decreased with time. As the blood volume in this tumor model is ~2% of the tumor volume, only ~15% of ^14^C label in the tumor will be in the blood pool. We estimated the functional vessel volume in this tumor previously using dynamic contrast agent‐enhanced MRI and by dye injection.^22^ A similar pattern of labeling was observed in the heart, which may be dominated by blood present in the ventricles. The decrease in blood activity was accompanied by small increases in all the other organs analyzed, including the brain. The total amount of radioactivity recovered from all the tissues analyzed was unchanged over 60 s (Figure 1).

**Figure 2:**
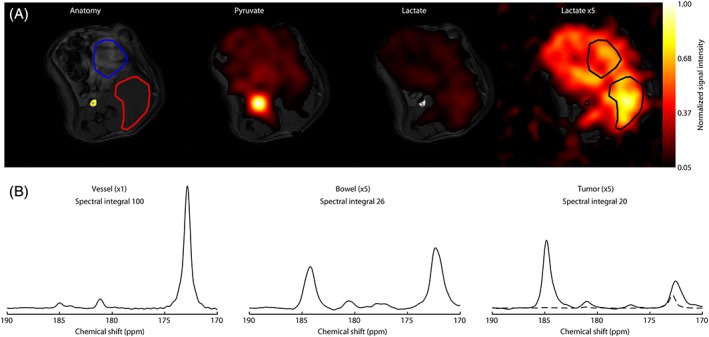
Images of pyruvate and lactate acquired 15 s after injection of hyperpolarized [1‐^13^C]pyruvate in a tumor‐bearing mouse (A). The grayscale image is an anatomical ^1^H image of tissue water. The blood vessel (yellow), tumor (red) and bowel (blue) regions are outlined. The false‐color images show the intensities of the pyruvate (172.9 ppm) and lactate (185.1 ppm) signals normalized to the maximum pyruvate signal in the slice. A lactate image from the same slice, multiplied by a factor of five, is also shown. Summed spectra from the blood vessel, bowel and tumor regions are shown below the corresponding images (B). The *y*‐scale for the bowel and tumor spectra has been multiplied by a factor of five to aid visualization. The broken line in the tumor spectrum shows the blood vessel spectrum, which has been scaled to take account of the fact that the blood volume is ~2% of the tumor volume

A ^13^C CSI acquired using a volume receiver coil at ~15 s after intravenous injection of hyperpolarized [1‐^13^C]pyruvate (10 mL/kg of 82mM) into a tumor‐bearing mouse showed that the pyruvate signal was predominantly in the aorta and that the signals from [1‐^13^C]lactate were distributed throughout the body. There was very little lactate signal in the aorta at this time point and only a small amount of pyruvate signal in the tumor (Figure 2). The pyruvate peak integral in the spectrum from the aorta was 14 times greater than the pyruvate peak integral in the spectrum from the tumor. Given a tumor blood volume of ~2%, the blood pyruvate signal will contribute ~30% of the total tumor pyruvate signal (see broken line in Figure 2B).

### 3.2 Concentrations of ^13^C‐labeled pyruvate and lactate in tumor and blood

The concentrations of ^13^C‐ and ^12^C‐labeled pyruvate and lactate were measured in blood and tumor tissue at 30 s after injection of [3‐^13^C]pyruvate (10 mL/kg, 82mM) (Table 1). The [3‐^13^C]pyruvate concentration in the tumor was undetectable, with all the observable label present in [3‐^13^C]lactate (0.78 μmol/g). Correcting for the presence of natural abundance ^13^C (1.1%) present in the lactate pool (0.19 μmol/g), this represents 4% of ID/g of ^13^C‐labeled pyruvate (16.4 μmol). This shows that, as soon as the labeled pyruvate enters the cell, the ^13^C label is rapidly diluted into the endogenous lactate pool in the exchange catalyzed by LDH.^17^, ^23^ The total tumor lactate concentration did not change significantly following the injection of pyruvate (Table 1), consistent with the observed isotope labeling being a result of the exchange of label rather than net conversion.^11^, ^24^, ^25^ The low value for %ID/g is consistent to some extent with the [1‐^14^C]pyruvate biodistribution data, which showed that, at 30 s, only ~1% of the ID was in the tumor. The ^14^C data represent label in both pyruvate and lactate. The sum of the ^13^C‐labeled pyruvate and lactate concentrations in the blood was 3.6 μmol/g (Table 1). Correcting for natural abundance ^13^C in lactate and pyruvate (0.2 μmol/g), this represents approximately 21% of the ID. Again, this is comparable with the [1‐^14^C]pyruvate biodistribution data (Figure 1), which showed that, at 30 s, there was approximately 15% of the injected ^14^C label in the blood. The lactate concentration was three times higher in the blood of tumor‐bearing mice (15 ± 3 μmol/g) when compared with controls (5.3 ± 2.1 μmol/g). The presence of a tumor has been shown previously to lead to an increase in blood lactate concentration.^26^ The blood [3‐^13^C]pyruvate and [3‐^13^C]lactate concentrations were 1.2 and 2.4 μmol/g, respectively, in tumor‐bearing mice, and 3.3 and 1.8 μmol/g, respectively, in non‐tumor‐bearing animals. These results show that there was significant labeling of blood lactate and that this increased at higher lactate concentrations. The increased labeling at high lactate concentrations is consistent with previous studies showing that increased lactate concentrations increase the rate of LDH‐catalyzed exchange of ^13^C label between pyruvate and lactate.^10^, ^11^, ^24^, ^27^ With a blood volume that is only 2% of the tumor volume, the labeled lactate and pyruvate concentrations in the blood pool (3.6 μmol/g) at 30 s will make only an ~10% contribution to the concentrations measured in the tumor (0.78 μmol/g). This is consistent with our failure to detect any ^13^C‐labeled pyruvate in the tumor (Table 1). To determine the relative contributions of blood cells and other tissues to the lactate labeling observed in the blood pool, we added 3.28 μmol of [3‐^13^C]pyruvate to 0.44 mL of freshly drawn blood obtained by cardiac puncture and measured labeling in pyruvate and lactate after incubation at 37°C for 30 s (Table 2). In blood from tumor‐bearing and non‐tumor‐bearing animals, 10% (1.2 μmol/g) and 9% (0.6 μmol/g), respectively, of the lactate became labeled (Table 2). Therefore, isotope exchange catalyzed by blood can account for approximately one‐half of the ^13^C‐labeled lactate observed in the blood pool at 30 s after injection of labeled pyruvate, the remainder presumably coming from ^13^C label exchange in other tissues.

**Table 1:** Total and ^13^C‐labeled pyruvate and lactate concentrations in blood and tumor

Mouse	Tissue	Total pyruvate concentration (μmol/g)	Total lactate concentration (μmol/g)	^13^C‐labeled pyruvate (% of total)	^13^C‐labeled lactate (% of total)
Tumor‐bearing	Blood (*n* = 7)	3.0 ± 1.2	15 ± 3 **^(^ a ^)^	40 ± 11**^(^ a ^)^	16 ± 3**^(^ a ^)^
	Tumor (*n* = 7)	n.q.	17 ± 3	–	4.6 ± 1.6
	Tumor with no [3‐^13^C]pyruvate injection (*n* = 3)	n.q	14 ± 4	–	–
Non‐tumor‐bearing	Blood (*n* = 6)	4.6 ± 2.4	5.3 ± 2.1	72 ± 11	33 ± 7

Mice were injected with 10 mL/kg of an 82mM solution of [3‐^13^C]pyruvate, except where indicated. Blood was obtained at 30 s by cardiac puncture and the tumors were immediately freeze‐clamped and extracted for ^13^C nuclear magnetic resonance (NMR) analysis of ^13^C labeling. Tumor samples from animals not injected with [3‐^13^C]pyruvate were taken and the blood was used to measure exchange in the blood pool (Table 2). n.q., not quantifiable; *n*, number of animals.

a Significantly different compared with levels in the blood of non‐tumor‐bearing mice; mean ± standard deviation (SD); *p* < 0.01.

**Table 2:** Exchange of ^13^C label between [3‐^13^C]pyruvate and endogenous lactate in blood

Source of blood	Total pyruvate concentration (μmol/g)	Total lactate concentration (μmol/g)	^13^C‐labeled pyruvate (% of total)	^13^C‐labeled lactate (% of total)
Tumor‐bearing mice (*n* = 3)	7 ± 3	12 ± 3	88 ± 2	10 ± 2
Non‐tumor‐bearing mice (*n* = 4)	7.8 ± 2.2	7 ± 3	92 ± 7	9 ± 2

[3‐^13^C]Pyruvate (3.28 μmol) was added to 0.44 mL of freshly withdrawn blood and incubated at 37°C for 30 s. The tissue was then extracted in perchloric acid and ^13^C labeling was analyzed by ^13^C nuclear magnetic resonance (NMR) measurements on neutralized extracts.

### 3.3 Compartmentalization of hyperpolarized [1‐^13^C]lactate

**Figure 3:**
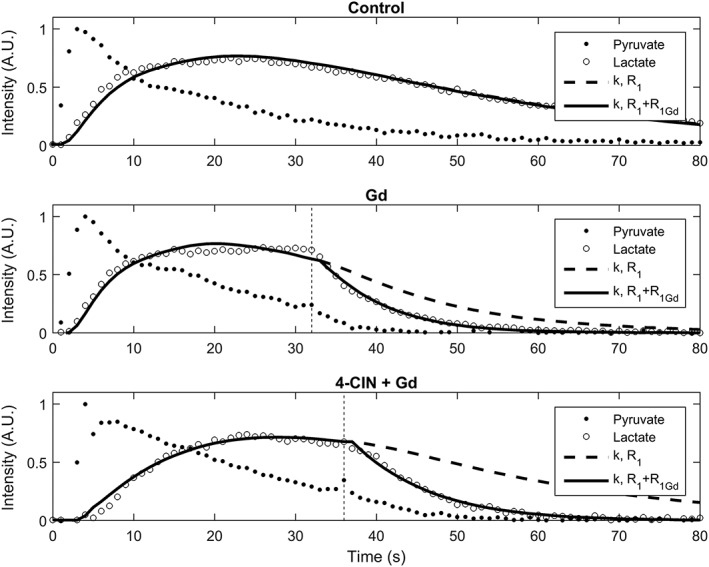
Effect of injection of a gadolinium chelate (Prohance) on the tumor lactate signal intensity. The contrast agent was injected ~35 s after injection of hyperpolarized [1‐^13^C]pyruvate at *t* = 0. The full line shows a fit to Equation (1). The broken line shows the expected lactate signal intensity if the gadolinium (Gd) chelate had not been injected. 4‐CIN, α‐cyano‐4‐hydroxycinnamate

Sequential injections of hyperpolarized [1‐^13^C]pyruvate (10 mL/kg, 82mM) (*n* = 4), followed ~35 s later by a gadolinium chelate (~0.75 mmol/kg Prohance), were used to investigate the distribution of labeled lactate between intra‐ and extracellular pools in the tumors.^18^, ^19^, ^28^ Previous reports have indicated that the rate of lactate transport to the extracellular compartment is only slightly slower than the rate of labeled lactate formation,^19^ and we have shown previously in EL4 tumor cells that transport and LDH activity have similar flux control coefficients for the exchange of hyperpolarized ^13^C label.^24^ Injection of the gadolinium chelate resulted in an increased rate of decay of the lactate signal, which could not be explained simply by the accelerated loss of pyruvate polarization (Figure 3). The broken lines in Figure 3 show the lactate signal that would have been observed based on the observed pyruvate signal and assuming that *k*_P_ does not change following the injection of the gadolinium chelate. This is consistent with some fraction of the lactate signal coming from the extracellular compartment, which will be predominantly the interstitial space, given the low blood volume. The administration of an MCT inhibitor (4‐CIN; 150 mg/kg, 0.2 mL intravenously), 3 min (*n* = 2) or 30 min (*n* = 4) prior to the injection of hyperpolarized [1‐^13^C]pyruvate (10 mL/kg, 82mM) (*n* = 6), led to a 30% decrease (*p* < 0.05) in the apparent first‐order rate constant (*k*_P_) describing the flux of hyperpolarized ^13^C label between the injected pyruvate and endogenous lactate pool (Table 3), consistent with 4‐CIN inhibition of pyruvate uptake.^17^, ^29^ This inhibitor, which inhibits predominantly MCT1, also inhibits lactate transport, which was confirmed by the injection of 4‐CIN 5 min prior to the injection of thermally polarized [1‐^13^C]lactate (10 mL/kg, 45mM) and measurement of labeled lactate concentrations in tumor extracts prepared 30 s later. There was an 85% decrease in the concentration of ^13^C‐labeled lactate measured in tumors treated with 4‐CIN (0.3 ± 0.1mM, *p* < 0.05, *n* = 2) when compared with controls treated with saline (2 ± 0.5mM, *n* = 3). There were no changes in the total tumor lactate concentration (12 ± 1mM). We have shown previously that EL4 tumors express both MCT1 and MCT4.^24^ The inhibition of lactate transport with 4‐CIN decreased the apparent rate of decay of polarization in [1‐^13^C]lactate from 0.056 ± 0.004 to 0.041 ± 0.003 s^–1^ (*p* < 0.05) (Table 3).

**Table 3:** Analysis of exchange rate constants and lactate polarization decay rates in tumors of untreated mice and mice treated with a monocarboxylate transporter (MCT) inhibitor (4‐CIN, α‐cyano‐4‐hydroxycinnamate). The animals were injected with a gadolinium chelate 35 s after injection of hyperpolarized [1‐^13^C]pyruvate

	*k*_P_ (s^–1^)	*R*_1_ (s^–1^)	*R*_1,Gd_ (s^–1^)
Control (*n* = 9)	0.153 ± 0.03	0.056 ± 0.004	0.091 ± 0.011^b^
4‐CIN (*n* = 6)	0.102 ± 0.014*	0.041 ± 0.003^a^	0.084 ± 0.005

The control group includes animals with (*n* = 4) and without (*n* = 5) gadolinium chelate injection. Lactate peak intensities were fitted to Equation (1).

a Different from control, *p* < 0.05 (Student's *t*‐test).

b The reported value is calculated only for animals receiving Prohance (*n* = 4). For control animals not receiving Prohance (*n* = 5), *R*_1,Gd_ was 0.001 ± 0.001 s^–1^.

## 4 DISCUSSION

The exchange of hyperpolarized ^13^C label between injected [1‐^13^C]pyruvate and the endogenous lactate pool is usually characterized by an apparent first‐order rate constant,^11^, ^17^, ^30^ or by the ratio of the area under the lactate and pyruvate labeling curves, which is directly related to this rate constant.^15^ The rate constant is an apparent rate constant as it is an empirical measure of a process that includes pyruvate delivery to the tissue, uptake into the cell and subsequent LDH‐catalyzed exchange of the hyperpolarized ^13^C label with lactate. As the rate of exchange is spatially heterogeneous,^11^ and in the absence of any imaging measurements, this apparent measured rate constant is also a reflection of the average rate for the whole tumor. However, it is not a measure of the metabolically relevant flux, measured for example in mM s^–1^, as a change in this apparent rate constant may indicate a change in flux or a change in the concentration of pyruvate at the exchange site. In order to obtain a true isotope flux measurement, we need to know the concentration of pyruvate at the exchange site, which measurements with hyperpolarized [1‐^13^C]pyruvate cannot readily provide.^8^, ^23^ The biodistribution studies presented here with [1‐^14^C]pyruvate show that, in tumor‐bearing animals, very little of the injected pyruvate reaches the tumor site; at 15 s, only 1.16% of the ID reaches the tumor. Measurements of pyruvate delivery to the tumor could not be performed with ^13^C‐labeled pyruvate as it was below the level of detection (Table 1). However, as measurements of ^13^C label in blood pyruvate and lactate (22% ID/g) were comparable with measurements of ^14^C label in the blood pool (15% ID/g), this suggests that the measurements of [1‐^14^C]pyruvate at the tumor site can provide a surrogate measure that can be used to approximate the concentration of ^13^C‐labeled pyruvate. Using the ^14^C data, we can calculate that, at 15 s following injection, 0.19 μmol/g of hyperpolarized [1‐^13^C]pyruvate would have reached the tumor site. With the ^13^C MRS measurements *in vivo*, which gave an apparent rate constant for the exchange of hyperpolarized ^13^C label between injected [1‐^13^C]pyruvate and lactate of 0.153 ± 0.03 s^–1^ (Table 3), this gives an isotope label flux of 0.030 μmol/s/g tumor. Measurements of [3‐^13^C]lactate concentration in extracts of tumors that were freeze‐clamped at 30 s after injection of thermally polarized [3‐^13^C]pyruvate (Table 1) can be used to estimate the true ^13^C label flux, which gave a flux of 0.026 μmol/s/g tumor. Given the likely errors introduced by tissue extraction, this is in remarkably good agreement with the flux estimated from the ^14^C and hyperpolarized ^13^C measurements, and demonstrates the internal consistency of the data. Potentially the same experiment could be performed clinically using a PET/MR machine with hyperpolarized [1‐^13^C]pyruvate^31^ and [1‐^11^C]pyruvate.^32^ However, the challenge would be to acquire the PET data sufficiently rapidly to obtain an estimate of the tumor pyruvate concentration during the time in which the MR measurements of hyperpolarized ^13^C label exchange were being made and before there was significant loss of ^11^C‐labeled material from the tissue. The estimates of label flux also show reasonably good agreement with previous measurements of ^13^C label flux between pyruvate and lactate in suspensions of EL4 cells *in vitro*.^24^ At 0.16mM [1‐^13^C]pyruvate, the label flux in EL4 tumor cell suspensions, estimated from the data shown by Witney et al.,^24^ is 3.5 fmol/min/cell. Assuming a tumor cell density *in vivo* of 5 × 10^8^ cells/mL,^33^ this corresponds to an extrapolated label flux in the tumor of 0.029 μmol/s/g tumor.

Previous diffusion measurements on EL4 tumors have shown that the labeled lactate observed following the injection of hyperpolarized [1‐^13^C]pyruvate has a lower diffusion coefficient than pyruvate, suggesting that the labeled lactate is predominantly intracellular.^17^ However, the reported diffusion coefficients in this previous study for pyruvate were slightly lower and for lactate slightly higher than those measured previously using ^1^H diffusion spectroscopy for purely vascular or intracellular metabolites, respectively,^34^, ^35^, ^36^ suggesting that the observed lactate was present in both the interstitial and intracellular spaces. That the tumor can release significant amounts of labeled lactate was confirmed here by the injection of a gadolinium chelate, Prohance, 35 s following the injection of hyperpolarized [1‐^13^C]pyruvate (Figure 3). The increased rate of decay of the lactate signal, which could not be explained by the accelerated decay of the [1‐^13^C]pyruvate polarization, demonstrated that lactate labeled intracellularly had been released into the extracellular space. The export of hyperpolarized ^13^C‐labeled lactate has been observed previously in studies on tumor cells *in vitro*.^25^, ^37^, ^38^ Further evidence for the release of labeled lactate in the tumor came from the effects of the inhibition of MCT activity with 4‐CIN. This resulted in a significant, although relatively small (~30%), decrease in the apparent rate of polarization decay in [1‐^13^C]lactate (*R*_1_) (Table 3), which will include contributions from spin–lattice relaxation and the export of lactate from the tumor. Assuming that MCT inhibition does not affect the spin–lattice relaxation, this again suggests that there was significant loss of labeled lactate from the tumor cell, which was inhibited by 4‐CIN. This modest decrease in [1‐^13^C]lactate *R*_1_, however, will have little effect on estimates of *k*_P_. We have shown previously that varying R_1_ for both [1‐^13^C]pyruvate and [1‐^13^C]lactate between 0.04 and 0.05 s^–1^ has only a small effect on the fitted value for *k*_P_ (see supplementary figure 2 in Day et al.^11^). The relatively rapid release of lactate from the tumor cells is interesting in view of the growing evidence for a symbiotic relationship between aerobic and hypoxic cancer cells within the same tumor, with lactate shuttling between these two cell populations.^39^ There has also been a recent suggestion that cancer cells with high glycolytic activity have a capacity to evade immunosurveillance by diminishing T‐cell anti‐tumor responses.^40^, ^41^

The inhibition of MCT activity by 4‐CIN was demonstrated by showing inhibition of lactate labeling following injection of hyperpolarized [1‐^13^C]pyruvate (Table 3) and also by showing the inhibition of tumor uptake of thermally polarized [1‐^13^C]lactate (10 mL/kg, 45mM). Interestingly, the latter experiments showed that much more of the labeled lactate than labeled pyruvate reached the tumor. Although there was no detectable ^13^C‐labeled pyruvate and only 0.78 μmol/g of ^13^C‐labeled lactate in the tumors of animals injected with 10 mL/kg of 82mM ^13^C‐labeled pyruvate (Table 1), which represents 4% of the injected pyruvate dose, there was ~2 μmol/g of ^13^C‐labeled lactate in animals injected with ^13^C‐labeled lactate. Correcting for natural abundance ^13^C in the lactate present in the tumor prior to the injection of labeled lactate, this corresponds to ~21% of the ID/g. This could be advantageous for experiments that use hyperpolarized ^13^C‐labeled lactate to measure LDH‐catalyzed exchange in tissues.^42^

Approximately 50% of the relatively high level of lactate labeling (2.4 μmol/g) observed in the blood of tumor‐bearing animals at 30 s following [3‐^13^C]pyruvate injection (67% ID/g) (Table 1) could be explained by erythrocyte‐catalyzed exchange, as incubation of [3‐^13^C]pyruvate in freshly drawn blood at 37°C for 30 s resulted in 1.2 μmol/g of labeled lactate (Table 2). The remainder of the labeled blood lactate presumably arose from lactate that underwent exchange in other tissues and was then released into the blood pool. The labeled lactate concentration at 30 s was approximately twice the concentration of ^13^C‐labeled pyruvate at this time point (1.2 μmol/g) (Table 1). CSIs acquired here, 15 s earlier (Figure 2), and a time course of single‐shot three‐dimensional images acquired previously at 2‐s intervals up to 60 s after hyperpolarized [1‐^13^C]pyruvate injection,^14^ showed that the majority of observable pyruvate was in blood; the intense signal in the aorta in Figure 2 should be noted, whereas labeled lactate was largely absent from the aorta and was more homogeneously distributed throughout the body, with a large fraction in the tumor. The implications of these observations are that labeled lactate released into the circulation at early time points is too low in concentration to be detected and at later time points has lost a substantial amount of polarization.

## 5 CONCLUSIONS

We have shown that only a small fraction of injected ^13^C‐labeled pyruvate reaches the tumor and the pyruvate that enters the cell rapidly exchanges its ^13^C label with lactate in the reaction catalyzed by LDH, the label becoming substantially diluted in the large endogenous lactate pool. Estimation of the pyruvate concentration in the tumor and multiplication of this concentration by the apparent first‐order rate constant (k_P_) describing the exchange of hyperpolarized ^13^C label between injected [1‐^13^C]pyruvate and the endogenous lactate pool gave a label flux that was in good agreement with that determined directly by measurement of the concentration of ^13^C label incorporated into lactate in tumor extracts. This suggests that simultaneous PET/MR studies in the clinic with hyperpolarized [1‐^13^C]pyruvate and [1‐^11^C]pyruvate could be used to estimate metabolically relevant isotope fluxes from the ^13^C data. There was measurable export of hyperpolarized ^13^C‐labeled lactate into the tumor extracellular space and the release of ^13^C‐labeled lactate from the tumor and from other tissues can explain the relatively high level of labeled lactate in the blood of animals injected with ^13^C‐labeled pyruvate. This labeled lactate was not detected in images acquired from animals injected with hyperpolarized [1‐^13^C]pyruvate, presumably because, at earlier time points, there was little labeled lactate in the blood and, at later time points, there will have been substantial loss of polarization.

## References

[1] Jadvar H , Alavi A , Gambhir SS . F‐^18^‐FDG uptake in lung, breast, and colon cancers: molecular biology correlates and disease characterization. J Nucl Med. 2009;50(11):1820‐1827. 19837767 10.2967/jnumed.108.054098 PMC2783751

[2] Gatenby RA , Gillies RJ . Why do cancers have high aerobic glycolysis? Nat Rev Cancer. 2004;4(11):891‐899. 15516961 10.1038/nrc1478

[3] Brindle K . New approaches for imaging tumour responses to treatment. Nat Rev Cancer. 2008;8(2):94‐107. 18202697 10.1038/nrc2289

[4] Ardenkjaer‐Larsen JH , Fridlund B , Gram A , et al. Increase in signal‐to‐noise ratio of >10,000 times in liquid‐state NMR. Proc Natl Acad Sci U S A. 2003;100(18):10158‐10163. 12930897 10.1073/pnas.1733835100 PMC193532

[5] Nelson SJ , Kurhanewicz J , Vigneron DB , et al. Metabolic imaging of patients with prostate cancer using hyperpolarized [1‐^13^C]pyruvate. Sci Transl Med. 2013;5(198):198ra108. https://doi.org/10.1126/scitranslmed.3006070 10.1126/scitranslmed.3006070 PMC4201045 23946197

[6] Cunningham CH , Lau JY , Chen AP , et al. Hyperpolarized ^13^C metabolic MRI of the human heart: initial experience. Circ Res. 2016;119(11):1177‐1182. 27635086 10.1161/CIRCRESAHA.116.309769 PMC5102279

[7] Brindle KM , Bohndiek SE , Gallagher FA , Kettunen MI . Tumor imaging using hyperpolarized ^13^C magnetic resonance spectroscopy. Magn Reson Med. 2011;66(2):505‐519. 21661043 10.1002/mrm.22999

[8] Brindle KM . Imaging metabolism with hyperpolarized ^13^C‐labeled cell substrates. J Am Chem Soc. 2015;137(20):6418‐6427. 25950268 10.1021/jacs.5b03300

[9] Albers MJ , Bok R , Chen AP , et al. Hyperpolarized ^13^C lactate, pyruvate, and alanine: noninvasive biomarkers for prostate cancer detection and grading. Cancer Res. 2008;68(20):8607‐8615. 18922937 10.1158/0008-5472.CAN-08-0749 PMC2829248

[10] Serrao EM , Kettunen MI , Rodrigues TB , et al. MRI with hyperpolarised [1‐^13^C]pyruvate detects advanced pancreatic preneoplasia prior to invasive disease in a mouse model. Gut. 2016;65:465‐475. 26347531 10.1136/gutjnl-2015-310114 PMC4789827

[11] Day SE , Kettunen MI , Gallagher FA , et al. Detecting tumor response to treatment using hyperpolarized ^13^C magnetic resonance imaging and spectroscopy. Nat Med. 2007;13(11):1382‐1387. 17965722 10.1038/nm1650

[12] Ward CS , Venkatesh HS , Chaumeil MM , et al. Noninvasive detection of target modulation following phosphatidylinositol 3‐kinase inhibition using hyperpolarized ^13^C magnetic resonance spectroscopy. Cancer Res. 2010;70(4):1296‐1305. 20145128 10.1158/0008-5472.CAN-09-2251 PMC2822895

[13] Brindle K . Watching tumours gasp and die with MRI: the promise of hyperpolarised ^13^C MR spectroscopic imaging. Br J Radiol. 2012;85(1014):697‐708. 22496072 10.1259/bjr/81120511 PMC3474112

[14] Wang J , Wright AJ , Hu DE , Hesketh R , Brindle KM . Single shot three‐dimensional pulse sequence for hyperpolarized ^13^C MRI. Magn Reson Med. 2017;77(2):740‐752. 26916384 10.1002/mrm.26168 PMC5297976

[15] Hill DK , Orton MR , Mariotti E , et al. Model free approach to kinetic analysis of real‐time hyperpolarized C‐13 magnetic resonance spectroscopy data. Plos One. 2013;8(9):9. 10.1371/journal.pone.0071996 PMC3762840 24023724

[16] Serrao EM , Rodrigues TB , Gallagher FA , et al. Effects of fasting on serial measurements of hyperpolarized 1‐C‐13 pyruvate metabolism in tumors. NMR Biomed. 2016;29(8):1048‐1055. 27309986 10.1002/nbm.3568 PMC4973679

[17] Kettunen MI , Kennedy BWC , Hu D‐E , Brindle KM . Spin echo measurements of the extravasation and tumor cell uptake of hyperpolarized [1‐^13^C]lactate and [1‐^13^C]pyruvate. Magn Reson Med. 2013;70(5):1200‐1209. 23280500 10.1002/mrm.24591

[18] Smith MR , Peterson ET , Gordon JW , et al. *In‐vivo* imaging and spectroscopy of dynamic metabolism using simultaneous (^13^)C and (^1^)H MRI. IEEE Trans Biomed Eng. 2012;59(1):45‐49. 21775254 10.1109/TBME.2011.2161988 PMC3676651

[19] Reineri F , Daniele V , Cavallari E , Aime S . Assessing the transport rate of hyperpolarized pyruvate and lactate from the intra‐ to the extracellular space. NMR Biomed. 2016;29(8):1022‐1027. 27271484 10.1002/nbm.3562

[20] Kazan SM , Reynolds S , Kennerley A , et al. Kinetic modeling of hyperpolarized C‐13 pyruvate metabolism in tumors using a measured arterial input function. Magn Reson Med. 2013;70(4):943‐953. 23169010 10.1002/mrm.24546

[21] Kettunen M , Kennedy B , Hu D‐E , Brindle K . Spin echo measurements of the extravasation and tumor cell uptake of hyperpolarized [1‐^13^C]lactate and [1‐^13^C]pyruvate. Magn Reson Med. 2012;70:1200‐1209. 23280500 10.1002/mrm.24591

[22] Hu D‐E , Beauregard DA , Bearchell MC , Thomsen LL , Brindle KM . Early detection of tumour immune‐rejection using magnetic resonance imaging. Br J Cancer. 2003;88(7):1135‐1142. 12671716 10.1038/sj.bjc.6600814 PMC2376373

[23] Xu T , Mayer D , Gu M , et al. Quantification of *in vivo* metabolic kinetics of hyperpolarized pyruvate in rat kidneys using dynamic ^13^C MRSI. NMR Biomed. 2011;24(8):997‐1005. 21538639 10.1002/nbm.1719 PMC3169748

[24] Witney TH , Kettunen MI , Brindle KM . Kinetic modeling of hyperpolarized ^13^C label exchange between pyruvate and lactate in tumor cells. J Biol Chem. 2011;286(28):24572‐24580. 21596745 10.1074/jbc.M111.237727 PMC3137032

[25] Harrison C , Yang C , Jindal A , et al. Comparison of kinetic models for analysis of pyruvate‐to‐lactate exchange by hyperpolarized ^13^C NMR. NMR Biomed. 2012;25(11):1286‐1294. 22451442 10.1002/nbm.2801 PMC3469722

[26] Argilés JM , López‐Soriano FJ . The energy state of tumor‐bearing rats. J Biol Chem. 1991;266(5):2978‐2982. 1993670

[27] Hurd RE , Spielman D , Josan S , Yen YF , Pfefferbaum A , Mayer D . Exchange‐linked dissolution agents in dissolution‐DNP ^13^C metabolic imaging. Magn Reson Med. 2013;70(4):936‐942. 23165935 10.1002/mrm.24544 PMC3660543

[28] Durst M , Koellisch U , Daniele V , et al. Probing lactate secretion in tumours with hyperpolarised NMR. NMR Biomed. 2016;29(8):1079‐1087. 27348729 10.1002/nbm.3574

[29] Harris T , Eliyahu G , Frydman L , Degani H . Kinetics of hyperpolarized ^13^C_1_‐pyruvate transport and metabolism in living human breast cancer cells. Proc Natl Acad Sci U S A. 2009;106(43):18131‐18136. 19826085 10.1073/pnas.0909049106 PMC2775348

[30] Zierhut ML , Yen Y‐F , Chen AP , et al. Kinetic modeling of hyperpolarized (^13^)C(_1_)‐pyruvate metabolism in normal rats and TRAMP mice. J Magn Reson (San Diego, Calif: 1997). 2010;202(1):85‐92. 10.1016/j.jmr.2009.10.003 PMC2833325 19884027

[31] Gutte H , Hansen AE , Larsen MM , et al. Simultaneous hyperpolarized ^13^C‐pyruvate MRI and ^18^F‐FDG PET (HyperPET) in 10 dogs with cancer. J Nucl Med. 2015;56(11):1786‐1792. 26338899 10.2967/jnumed.115.156364

[32] Hara T , Iio M , Izuchi R , Tsukiyama T , Yokoi F . Synthesis of pyruvate‐1‐^11^C as a radiopharmaceutical for tumor imaging. Eur J Nucl Med. 1985;11(6–7):275‐278. 4076234 10.1007/BF00279083

[33] Artemov D , Mori N , Ravi R , Magnetic BZM . resonance molecular imaging of the HER‐2/neu receptor. Cancer Res. 2003;63(11):2723‐2727. 12782573

[34] Nicolay K , Braun KP , Graaf RA , Dijkhuizen RM , Kruiskamp MJ . Diffusion NMR spectroscopy. NMR Biomed. 2001;14(2):94‐111. 11320536 10.1002/nbm.686

[35] Pfeuffer J , Flogel U , Dreher W , Leibfritz D . Restricted diffusion and exchange of intracellular water: theoretical modelling and diffusion time dependence of ^1^H NMR measurements on perfused glial cells. NMR Biomed. 1998;11(1):19‐31. 9608585 10.1002/(sici)1099-1492(199802)11:1<19::aid-nbm499>3.0.co;2-o

[36] Sotak CH . Multiple quantum NMR spectroscopy methods for measuring the apparent self‐diffusion coefficient of in vivo lactic acid. NMR Biomed. 1991;4(2):70‐72. 1859787 10.1002/nbm.1940040207

[37] Koelsch BL , Sriram R , Keshari KR , et al. Separation of extra‐ and intracellular metabolites using hyperpolarized (^13^)C diffusion weighted MR. J Magn Reson. 2016;270:115‐123. 27434780 10.1016/j.jmr.2016.07.002 PMC5448422

[38] Keshari KR , Sriram R , Koelsch BL , et al. Hyperpolarized ^13^C‐pyruvate magnetic resonance reveals rapid lactate export in metastatic renal cell carcinomas. Cancer Res. 2013;73(2):529‐538. 23204238 10.1158/0008-5472.CAN-12-3461 PMC3548990

[39] Sonveaux P , Vegran F , Schroeder T , et al. Targeting lactate‐fueled respiration selectively kills hypoxic tumor cells in mice. J Clin Invest. 2008;118(12):3930‐3942. 19033663 10.1172/JCI36843 PMC2582933

[40] Ho P‐C , Bihuniak JD , Macintyre AN , et al. Phosphoenolpyruvate is a metabolic checkpoint of anti‐tumor T cell responses. Cell. 2015;162(6):1217‐1228. 26321681 10.1016/j.cell.2015.08.012 PMC4567953

[41] Chang CH , Qiu J , O'Sullivan D , et al. Metabolic competition in the tumor microenvironment is a driver of cancer progression. Cell. 2015;162(6):1229‐1241. 26321679 10.1016/j.cell.2015.08.016 PMC4864363

[42] Kennedy BWC , Kettunen MI , Hu D‐E , Brindle KM . Probing lactate dehydrogenase activity in tumors by measuring hydrogen/deuterium exchange in hyperpolarized L‐[1‐^13^C,U‐^2^H]lactate. J Am Chem Soc. 2012;134:4969−4977. 22316419 10.1021/ja300222e PMC3303201

